# Supplementary material for: The American Cherimoya Genome Reveals Insights into the Intra-Specific Divergence, the Evolution of Magnoliales, and a Putative Gene Cluster for Acetogenin Biosynthesis
Source: Plants (Basel). 2024 Feb 26;13(5):636. doi: 10.3390/plants13050636 (PMC10934984; doi:10.3390/plants13050636)
Supplement: Supplementary file 1 [file plants-13-00636-s001.zip › plants-2855821-supplementary.pdf]

Supplementary figures

The American cherimoya genome reveals insights into the intra-specific divergence, the evolution of *Magnoliales*, and a putative gene cluster for acetogenin biosynthesis

Tang Li[1*], Jinfang Zheng[1*], Orestis Nousias[1], Lyndel W Meinhardt[2] , Ricardo Goenaga[3] Dapeng Zhang[2,#], Yanbin Yin[1,#]

[1]Nebraska Food for Health Center, Department of Food Science and Technology, University of Nebraska, Lincoln, NE 68588, USA
[2]USDA-ARS, Sustainable Perennial Crops Laboratory, Beltsville, MD 20705, USA
[3]USDA-ARS, Tropical Agriculture Research Station, Mayaguez, PR 00680

**co-first authors*
[#]corresponding authors

Yanbin Yin
Tel: 1-402-472-4303
Email: yyin@unl.edu

Dapeng Zhang
Tel: 1-301-504-7477
Email: dapeng.zhang@usda.gov

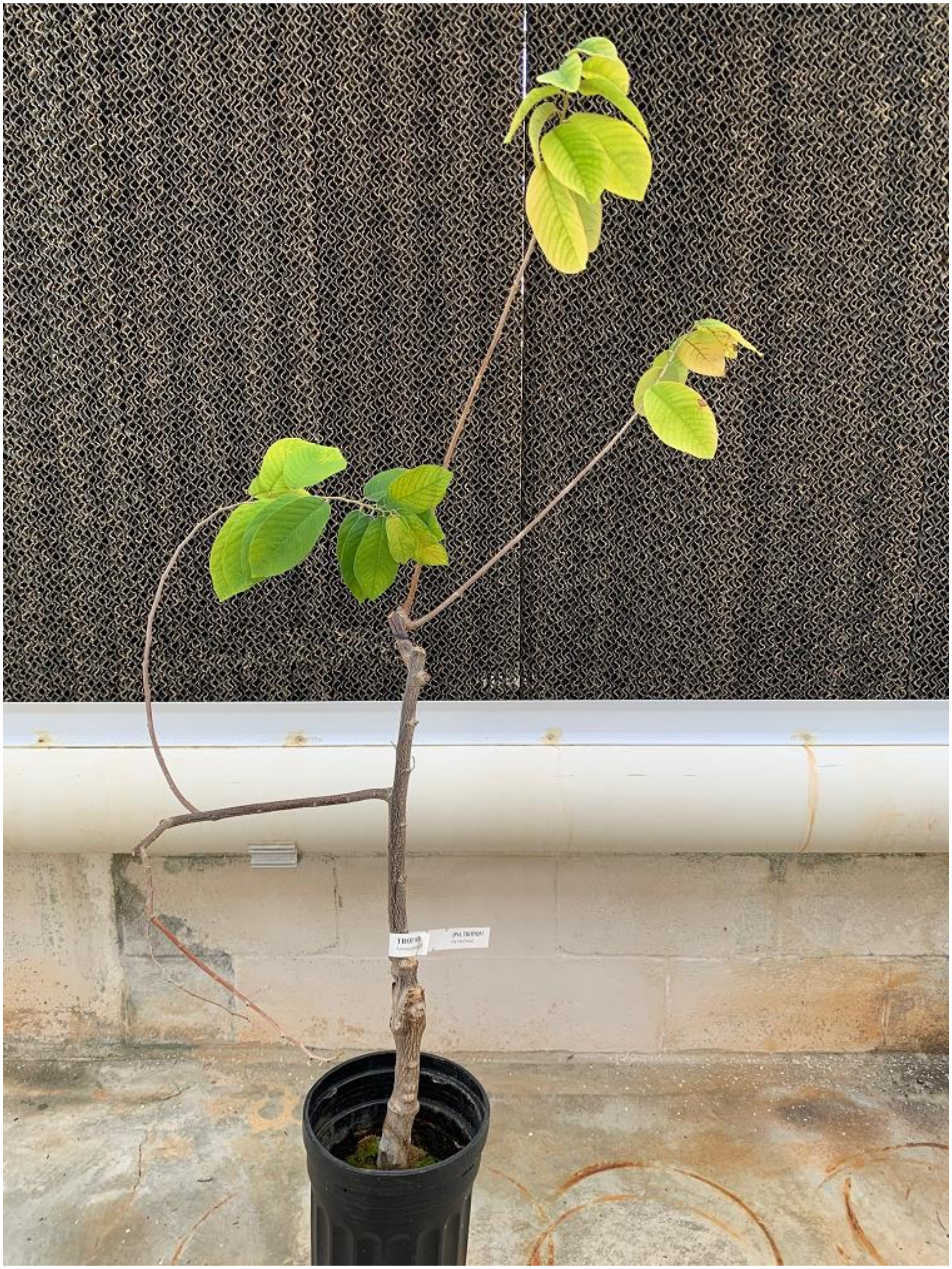

Figure S1. The young plant of cherimoya cultivar ‘Booth’ in the greenhouse of USDA-ARS, Sustainable Perennial Crops Laboratory, Beltsville, Maryland.

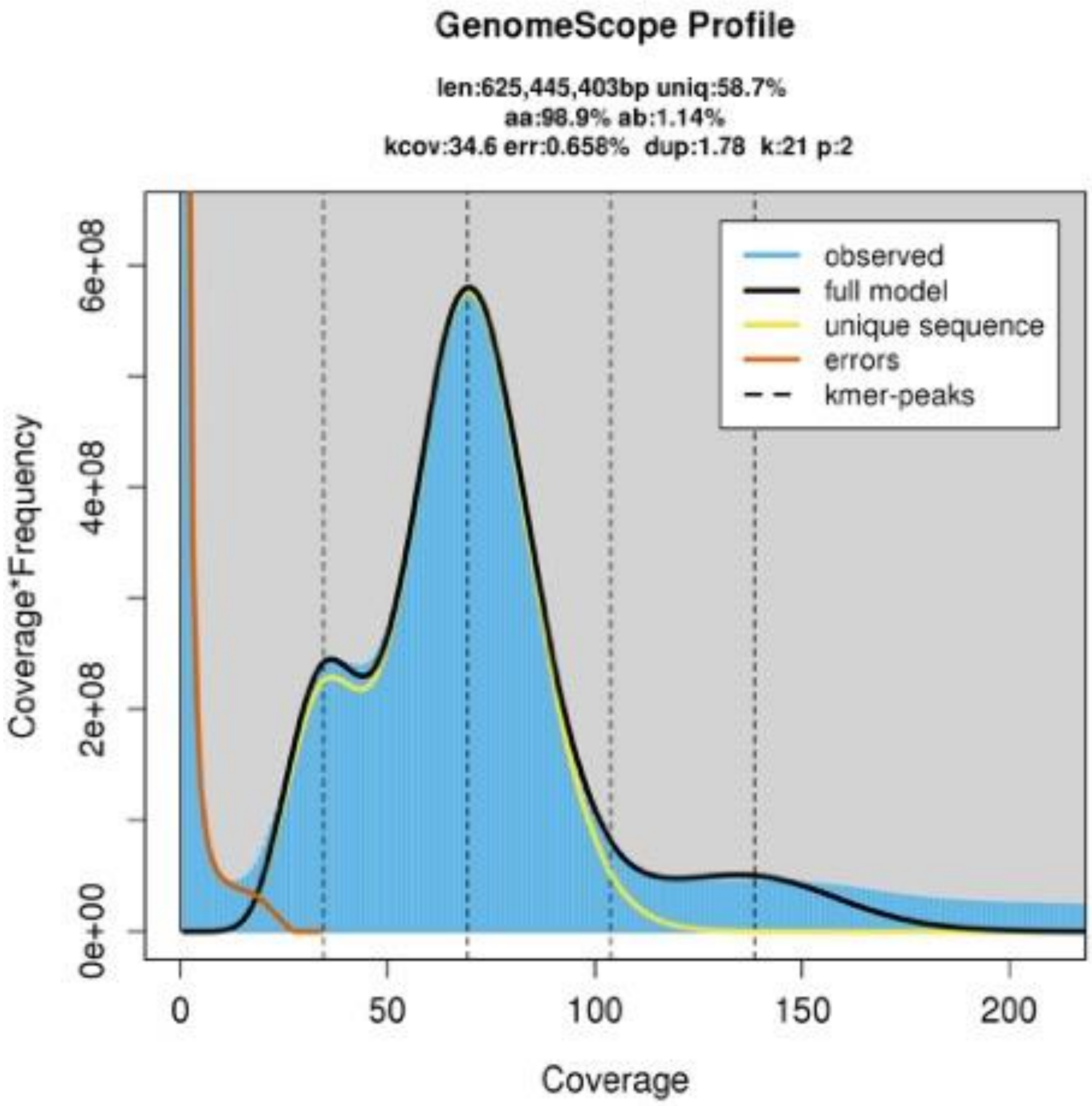

Figure S2. The k-mer frequency plot for *A. cherimola* genome.

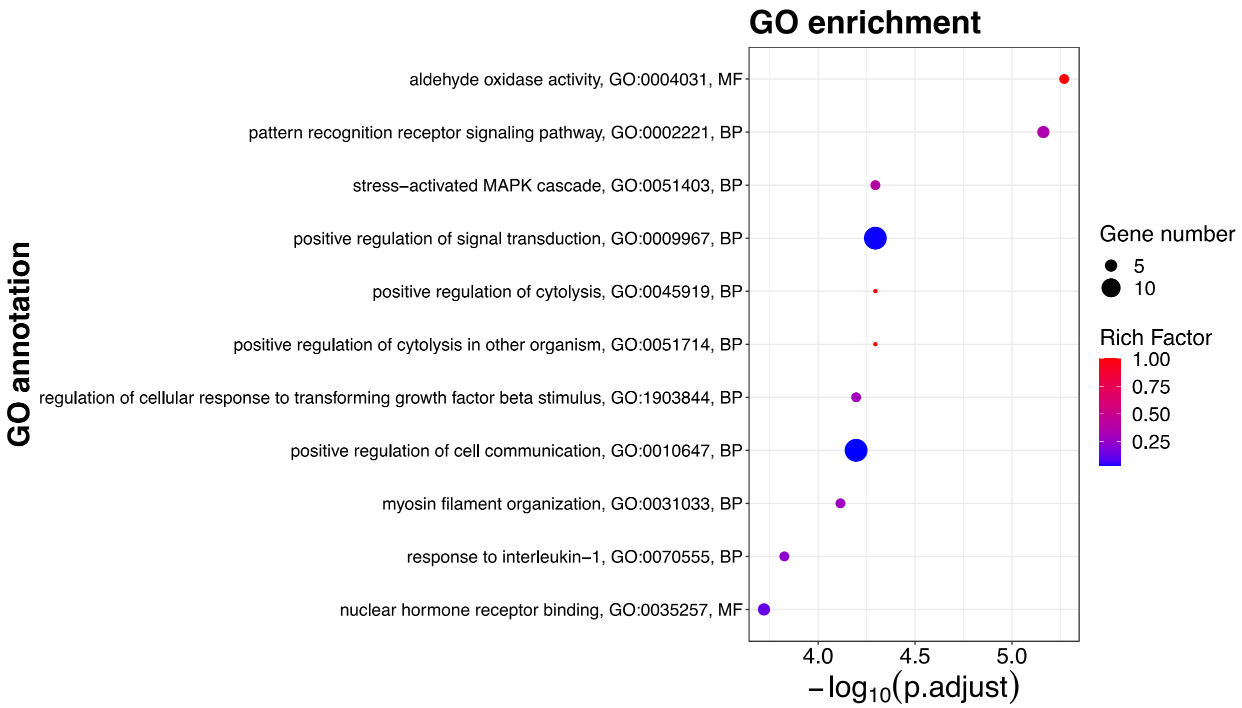

Figure S3. The GO enrichment for 51 significantly contracted gene families in *A. cherimola*. See **Figure 3B** for legends.

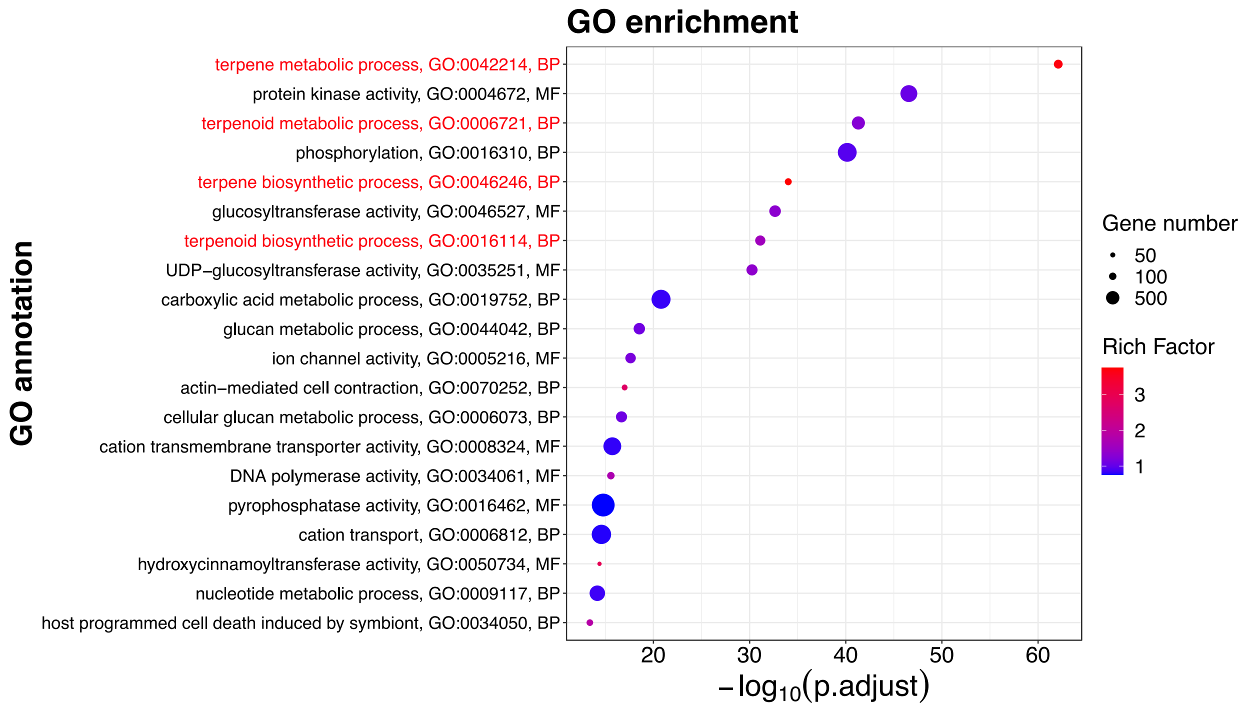

Figure S4. The top 20 GO enrichment of 11,698 unique OGCs in magnoliids clade. The terpene/terpenoids synthesis and metabolism functions are enriched in magnoliid species. See **Figure 3B** for legends.

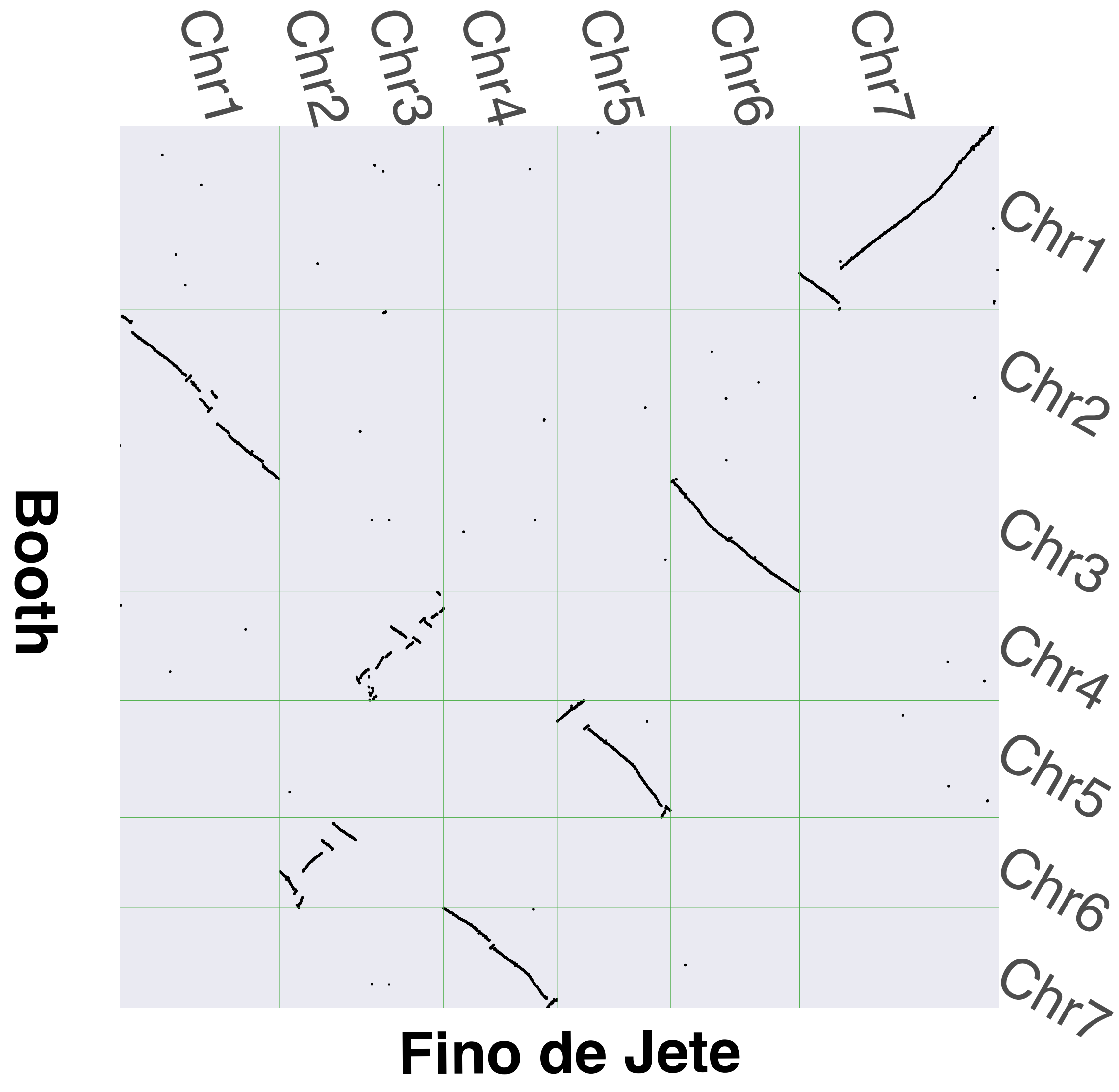

Figure S5. Syntenic block plot of genes in **"Fino de Jete" and "Booth"** genomes computed by JCVI (https://github.com/tanghaibao/jcvi). Only the seven chromosomes of the two genome assemblies were aligned. The "Booth" chromosomes (y-axis) are numbered according to the Chr length, while the "Fino de Jete" chromosomes (x-axis) are not. Each dot in the plot is a gene syntenic block.
